# The Predictive and Prognostic Value of the Systemic Immune-Inflammation Index for Congestive Heart Failure

**DOI:** 10.31083/j.rcm2511417

**Published:** 2024-11-21

**Authors:** Zhihao Zheng, Shanshan Shi, Zechen Liu, Yanjun Song, Zhen’ge Chang, Kongyong Cui, Chenxi Song, Jia Li, Kefei Dou

**Affiliations:** ^1^Cardiometabolic Medicine Center, Fuwai Hospital, National Center for Cardiovascular Diseases, Chinese Academy of Medical Sciences and Peking Union Medical College, 100037 Beijing, China; ^2^State Key Laboratory of Cardiovascular Disease, 102308 Beijing, China; ^3^National Clinical Research Center for Cardiovascular Diseases, 100101 Beijing, China; ^4^Department of Respiratory Medicine, Civil Aviation General Hospital, 100123 Beijing, China

**Keywords:** systemic immune-inflammation index, heart failure, inflammation, NHANES, prediction

## Abstract

**Background:**

The systemic immune-inflammatory index (SII), calculated by (platelet count × neutrophil count)/lymphocyte count, is a novel biomarker with predive and prognostic value in numerous diseases. However, the relationship between SII and congestive heart failure (CHF) is not clear. This study aims to document the association of SII with the prevalence of CHF in the whole population and the long-term prognosis in CHF patients.

**Methods:**

This study included 57,500 participants in the National Health and Nutritional Examination Surveys, who were categorized into 3 categories based on their SII levels. A cross-sectional study was conducted to examine the relationship between SII and CHF prevalence in the whole population, followed by a prospective longitudinal study with a 5.4-year follow-up period for CHF patients to assess the predictive significance of SII for CHF. The main focus of the longitudinal study was on all-cause death as the primary outcome, with cardiovascular (CV) death as the secondary outcome. Associations were estimated using multivariate logistic regression and Cox proportional hazards models. The dose-response relationship was assessed with the restricted cubic spline (RCS) analysis.

**Results:**

In the cross-sectional analysis, there were 1927 (3.35%) participants diagnosed with CHF. The high SII group showed a significantly higher prevalence of CHF than the low SII group (odds ratio (OR) 1.24, 95% confidence interval (CI): 1.05, 1.45). In the longitudinal analysis, 882 all-cause deaths including 379 CV deaths were collected among CHF patients, and high SII was associated with a significant increase in the risk of all-cause death (hazard ratio (HR) 1.44; 95% CI: 1.14, 1.81) and CV death (HR 1.31; 95% CI: 1.08, 1.58). RCS confirmed the positive correlation of SII with the prevalence of CHF in the whole population, as well as the mortality risk in CHF patients.

**Conclusions:**

This study is the first to reveal that high SII was related to a high prevalence of CHF and a poor prognosis in CHF patients. These findings underscore the potential role of SII in the prevention and management of CHF.

## 1. Introduction

Congestive heart failure (CHF) presents with left or right ventricular dysfunction which results in insufficient output for perfusion of tissues and organs [1]. CHF has been regarded as a major clinical and public health problem due to its high prevalence and poor prognosis [2]. There are more than 64.3 million patients with CHF worldwide, and the prevalence of CHF ranges from 1% to 12% based on United States and European studies [2, 3, 4]. Statistical reports showed that 30-day CHF case mortality ranges from 4.5–8.6%, and 1-year mortality ranges from 4% to 45%, averaging 33% [3]. Therefore, precise evaluation for populations with a high risk for CHF and prognostic assessment for patients with CHF need to be investigated.

Numerous studies have documented that a high inflammatory burden plays a critical role in the pathogenesis and progress of CHF [5]. In recent studies, inflammatory receptors such as toll-like receptors and the activated downstream pro-inflammatory signaling factors such as NF-κB (nuclear factor kappa-light-chain-enhancer of activated B cell) are key contributors to the pathogenesis of CHF by increasing the production of inflammatory cytokines such as interleukin 6 and tumor necrosis factor [6]. Cardiomyocyte hypertrophy, apoptosis, and fibrosis are subsequently induced by the increased inflammation and contribute to cardiac remodeling, which further decreases cardiac function [7]. Therefore, the role of inflammatory biomarkers in predicting the incidence and outcomes in patients with CHF is of great interest.

The systemic immune-inflammation index (SII) is a novel composite inflammatory biomarker that combines three important immune cells: platelets, lymphocytes, and neutrophils. Patients with an elevated SII usually have thrombocytopenia, neutrophilia, or lymphopenia, suggesting an elevated inflammatory status and weak immune response [8]. The predictive and/or prognostic value of SII has been determined in numerous diseases such as coronary heart disease (CHD) [9], stroke [10], cancers [11], and hepatic steatosis [12]. Studies have shown that high a SII is associated with negative outcomes in critically ill patients with CHF [13, 14]. However, this finding limited to patients who were hospitalized in the intensive care unit (ICU) and needs to be validated for all CHF patients. Furthermore, the correlation between SII and the incidence of CHF in the whole population has not been previously investigated.

Because of these limitations, we used the U.S. National Health and Nutrition Examination Survey (NHANES) to conduct a cross-sectional study with 50,000 participants to determine the association of SII with the prevalence of CHF; along with a longitudinal study with 5000 patients with CHF to assess the impact of SII on the prognosis of patients with CHF.

## 2. Methods

### 2.1 Study Design and Participants

**Fig. 1.**
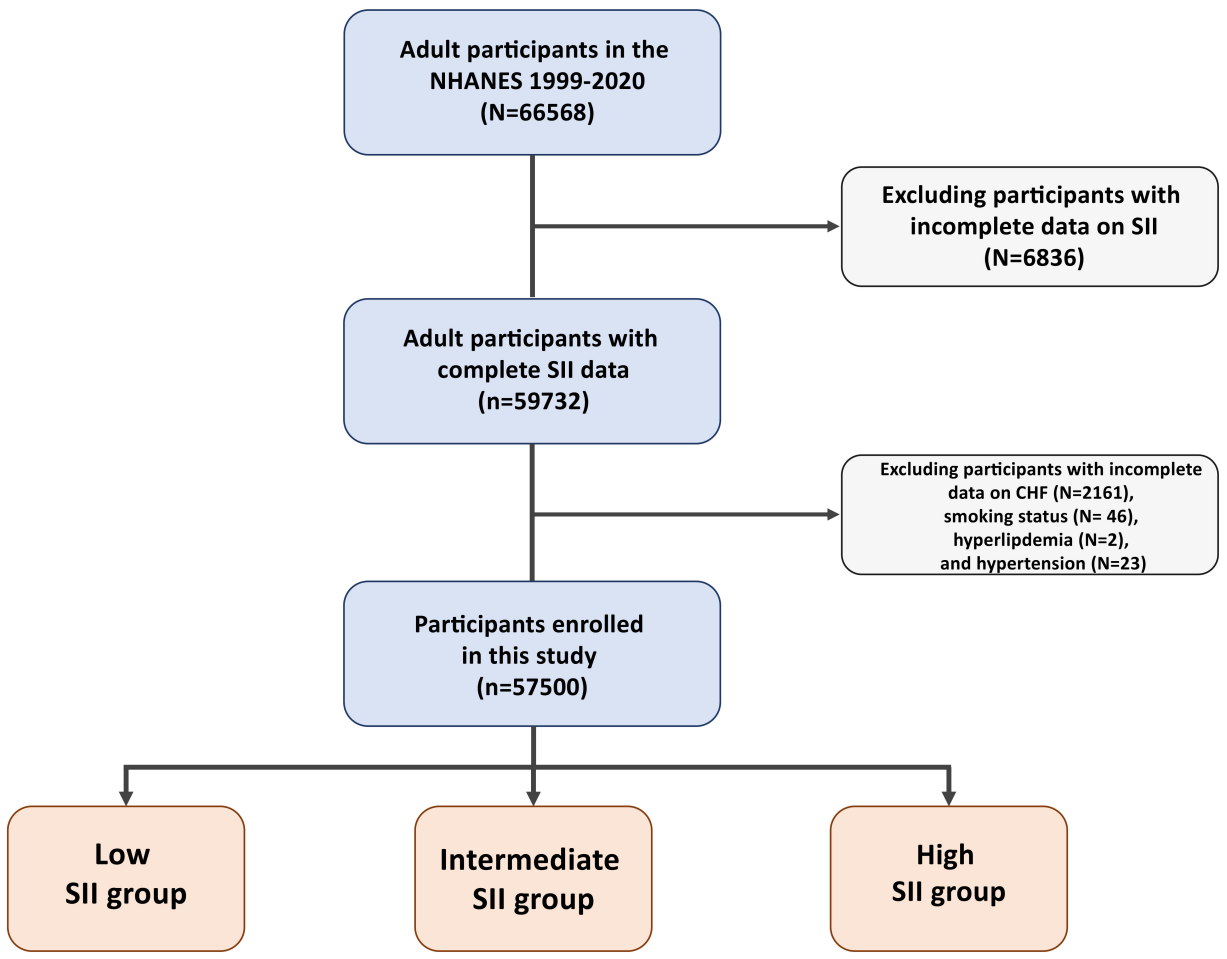
**Flow chart of the current study**. Abbreviations: CHF, congestive heart failure; SII, systemic immune-inflammation index; NHANES, National Health and Nutrition Examination Survey.

The current study utilized data from NHANES from 1999–2020. The exclusion criteria included a lack of information on SII, a CHF diagnosis, and cardiovascular disease (CVD) risk factors. After excluding participants with incomplete SII data (n = 6836), heart failure diagnosis (n = 2161), smoking status (n = 46), hyperlipemia (n = 2), and hypertension (n = 23), we ultimately analyzed 57,500 participants in the final analysis (Fig. 1, **Supplementary Fig. 1**). Participants were distributed into 3 categories according to the tertiles of SII: groups with SII-low (<383.9 in the whole population, <407.0 in CHF patients), SII- medium (383.9–596.7 in the whole population, 407.0–672.8 in CHF patients), and SII-high (>596.8 in the whole population, >672.8 in CHF patients). We first performed a cross-sectional analysis of the whole population to determine the association between SII levels and the prevalence of CHF. Next, we performed a prospective longitudinal analysis with a clinical follow-up in patients with CHF to investigate the predictive value of SII for the outcomes of all-cause and cardiovascular (CV) death.

### 2.2 Exposure Variable

The exposure variable of this study is SII. SII was calculated from the formula: (platelet count × neutrophil count)/lymphocyte count [8]. Platelet, neutrophil, and lymphocyte counts (expressed as ×10^3^ cells/mL) were measured using automated hematology analyzers.

### 2.3 Definitions

CHF was diagnosed based on the Monetary Choice Questionnaire: the question “Have you ever been told you had congestive heart failure?” Or “Has a doctor or other health professional ever told you that you had a heart failure?” [15]. The diagnosis criteria of atherosclerotic cardiovascular disease (ASCVD) included CHD, angina, heart attack, and stroke. Diabetes mellitus, hyperlipidemia, and hypertension were identified using the guidelines from previous literature sources [16, 17].

### 2.4 Outcomes and Follow-Up

CHF was the dependent variable in the cross-sectional study. This study examines the long-term results, focusing on all-cause death and CV death. To determine mortality status, death certificates linked to the National Death Index were examined through December 31, 2019. Causes of death were classified according to the International Statistical Classification of Disease, Tenth Revision (ICD-10, heart diseases: I00–I09, I11, I13, I20–I51; cerebrovascular diseases: I60–I69).

In the cohort study, we conducted a clinical follow-up for patients with CHF, with a median follow-up duration of 5.4 years. The duration of follow-up was calculated from the NHANES Mobile Examination Center (MEC) date until the date of death or the conclusion of follow-up (December 31, 2019), whichever came first.

### 2.5 Covariates

Information on demographic characteristics and self-reported medical conditions was collected via standardized questionnaires administered by trained interviewers during in-home interviews. Physical examinations were conducted at the MEC following standard protocols to collect body measurements and blood sample data.

Education levels were divided into under high school, high school/equivalent, and college/higher. Race/ethnicity categories included non-Hispanic White, non-Hispanic Black, Mexican American, and others. Physical activity was quantified by weekly minutes of activities multiplied by the metabolic equivalent (MET, minutes/week) level, categorized into sedentary (MET = 0, without regular physical activity), insufficient (MET 0–500), moderate (MET 500–1000), and high (>1000 MET) [17]. The family income-to-poverty ratio (PIR) was categorized into three groups: ≤1.0, 1.0–3.0, and >3.0. Smoking status was classified as never (<100 cigarettes during lifetime), former (≥100 cigarettes during lifetime, quit smoking), and current (≥100 cigarettes during lifetime, still smoking). Body mass index (BMI) was calculated by dividing weight in kilograms by height in meters squared.

### 2.6 Statistical Analysis

To ensure a sample representative of the US national population, we utilized suitable weights as per the NHANES complex sampling design. Baseline characteristics were reported as frequency (weighted percentages) for categorical variables and weighted means ± standard error for continuous variables. Group differences at baseline were assessed using χ^2^ tests for categorical variables and analysis of variance (ANOVA) for continuous variables. The percentages of missing data for covariates was below 5% (BMI [1.32%]). Missing values for family income-to-poverty (9.4%) were assigned to a separate “Unknown” category. Imputation with the median of each variable was employed to include all data in the modeling.

Odd ratios (ORs) and 95% confidence intervals (CIs) for the association between SII and CHF prevalence in the whole population were estimated using multivariate logistic regression models. Hazard ratio (HR) and 95% CIs for the association between SII and the risk of all-cause and CV death were calculated using multivariate Cox regression models. Kaplan-Meier (K-M) plots were performed for survival analysis, with statistical significance determined by the Log-rank test. For continuous variable analysis, SII was log-transformed. To estimate the dose-response relationship between SII and the risk of CHF and death, restricted cubic spline (RCS) analysis with 4 knots (5th, 35th, 65th, and 95th percentiles) was performed in the fully adjusted model. Nonlinearity was tested using the likelihood ratio test. Receiver operating characteristic (ROC) curves were plotted, the area under the curve (AUC) and Youden’s index were calculated to evaluate the predictive performance of SII for the prognosis of CHF patients.

In the regression analysis, we progressively adjusted for potential covariates across three models. Model 1 was adjusted for age, sex, and race/ethnicity. Model 2 was further adjusted for smoking status, physical activity, education levels, PIR, and BMI. Model 3 was further adjusted for ASCVD, diabetes, hyperlipidemia, and hypertension.

Subgroup analyses were conducted according to corrected variables, and multiplicative interaction terms were included to assess interactions. Sensitivity analyses were performed after excluding non-Hispanic Black participants, those with missing BMI, PIR data, and those who died within 90 days of the follow-up period.

All analyses were performed with R version 4.1.3 (R Foundation for Statistical Computing, Vienna, Austria). A 2-tailed *p*-value < 0.05 was considered significant.

## 3. Results

### 3.1 Baseline Characteristics

Table 1 shows the baseline characteristics of the whole population grouped by SII levels. The overall weighted mean age was 47.46 years and 48.12% were male. Participants with high SII levels were older than those with low SII levels and tended to be non-Hispanic white people, smokers, have lower levels of education, family income, and physical activity, and were more likely to have combinations of ASCVD, diabetes, hyperlipidemia, hypertension, and CHF. **Supplementary Table 1**, sows the baseline characteristics of participants with and without CHF. Patients with CHF had much higher SII levels than those without CHF. The baseline features of CHF patients grouped by SII levels are presented in **Supplementary Table 2**.

**Table 1.** **Baseline characteristics of the whole participants based on the SII in NHANES**.

Characteristics		Total (N = 57,500)	SII			*p* value
			Low (N = 19,168)	Median (N = 19,165)	High (N = 19,167)	
Age (years)		47.46 ± 0.19	46.72 ± 0.25	47.36 ± 0.21	48.27 ± 0.24	<0.001
Sex, n (%)						<0.001
	Male	27,667 (48.12)	10,244 (53.87)	9251 (48.25)	8172 (42.25)	
	Female	29,833 (51.88)	8924 (46.13)	9914 (51.75)	10,995 (57.75)	
Race/ethnicity, n (%)						<0.001
	Non-Hispanic White people	24,853 (43.22)	6470 (60.37)	8600 (69.11)	9783 (72.40)	
	Non-Hispanic Black people	12,151 (21.13)	5912 (16.53)	3432 (8.91)	2807 (7.51)	
	Mexican American people	9676 (16.83)	2910 (8.58)	3393 (8.46)	3373 (8.20)	
	Others	10,820 (18.82)	3876 (14.52)	3740 (13.52)	3204 (11.88)	
Education level, n (%)						<0.001
	Less than high school	14,892 (25.9)	5035 (16.41)	4942 (15.29)	4915 (15.96)	
	High school or equivalent	13,367 (23.25)	4298 (22.64)	4423 (24.28)	4646 (25.28)	
	College or above	29,241 (50.85)	9835 (60.95)	9800 (60.43)	9606 (58.77)	
Family income to poverty ratio, n (%)						0.003
	<1	10,694 (18.6)	3543 (12.94)	3512 (12.58)	3639 (13.34)	
	≥1 & <3	21,976 (38.22)	7288 (33.43)	7134 (31.86)	7554 (34.37)	
	≥3	19,434 (33.8)	6438 (46.11)	6755 (48.13)	6241 (44.95)	
	Unknown	5396 (9.38)	1899 (7.52)	1764 (7.43)	1733 (7.34)	
Smoking status, n (%)						<0.001
	Never	31,566 (54.9)	10,921 (57.26)	10,621 (55.58)	10,024 (51.56)	
	Former	14,185 (24.67)	4611 (24.85)	4661 (24.30)	4913 (25.36)	
	Current	11,749 (20.43)	3636 (17.89)	3883 (20.12)	4230 (23.08)	
BMI (kg/m^2^), n (%)						<0.001
	<25.0	16,378 (28.48)	5785 (32.51)	5327 (28.40)	5266 (27.71)	
	25.0–29.9	19,971 (34.73)	6853 (35.20)	6730 (34.43)	6388 (31.90)	
	≥30.0	21,151 (36.78)	6530 (32.28)	7108 (37.17)	7513 (40.40)	
Physical activity, n (%)						<0.001
	Sedentary	15,904 (27.66)	4921 (19.52)	5147 (21.36)	5836 (24.93)	
	Insufficient	11,877 (20.66)	3547 (16.34)	3986 (18.53)	4344 (21.03)	
	Moderate	6566 (11.42)	2105 (10.79)	2235 (11.64)	2226 (12.43)	
	High	23,153 (40.27)	8595 (53.35)	7797 (48.46)	6761 (41.61)	
Diabetes, n (%)		10,287 (17.89)	3371 (12.58)	3319 (13.43)	3597 (15.69)	<0.001
Hyperlipidemia, n (%)		40,912 (71.15)	12,981 (65.61)	13,899 (71.48)	14,032 (72.13)	<0.001
Hypertension, n (%)		23,837 (41.46)	7711 (33.97)	7778 (36.01)	8348 (40.62)	<0.001
CHF, n (%)		1927 (3.35)	575 (2.17)	553 (2.00)	799 (3.18)	<0.001
ASCVD, n (%)		5910 (10.28)	1841 (7.95)	1855 (7.67)	2214 (9.10)	0.001

Data are presented as weighted means ± SEs for continuous variables and unweighted numbers (weighted percentages) for categorical variables. Abbreviations: ASCVD, atherosclerotic cardiovascular disease; BMI, body mass index; CHF, congestive heart failure; SII, systemic immune-inflammation index; NHANES, National Health and Nutrition Examination Survey.

### 3.2 Cross-Sectional Analysis: SII and the Incidence of CHF

**Fig. 2.**
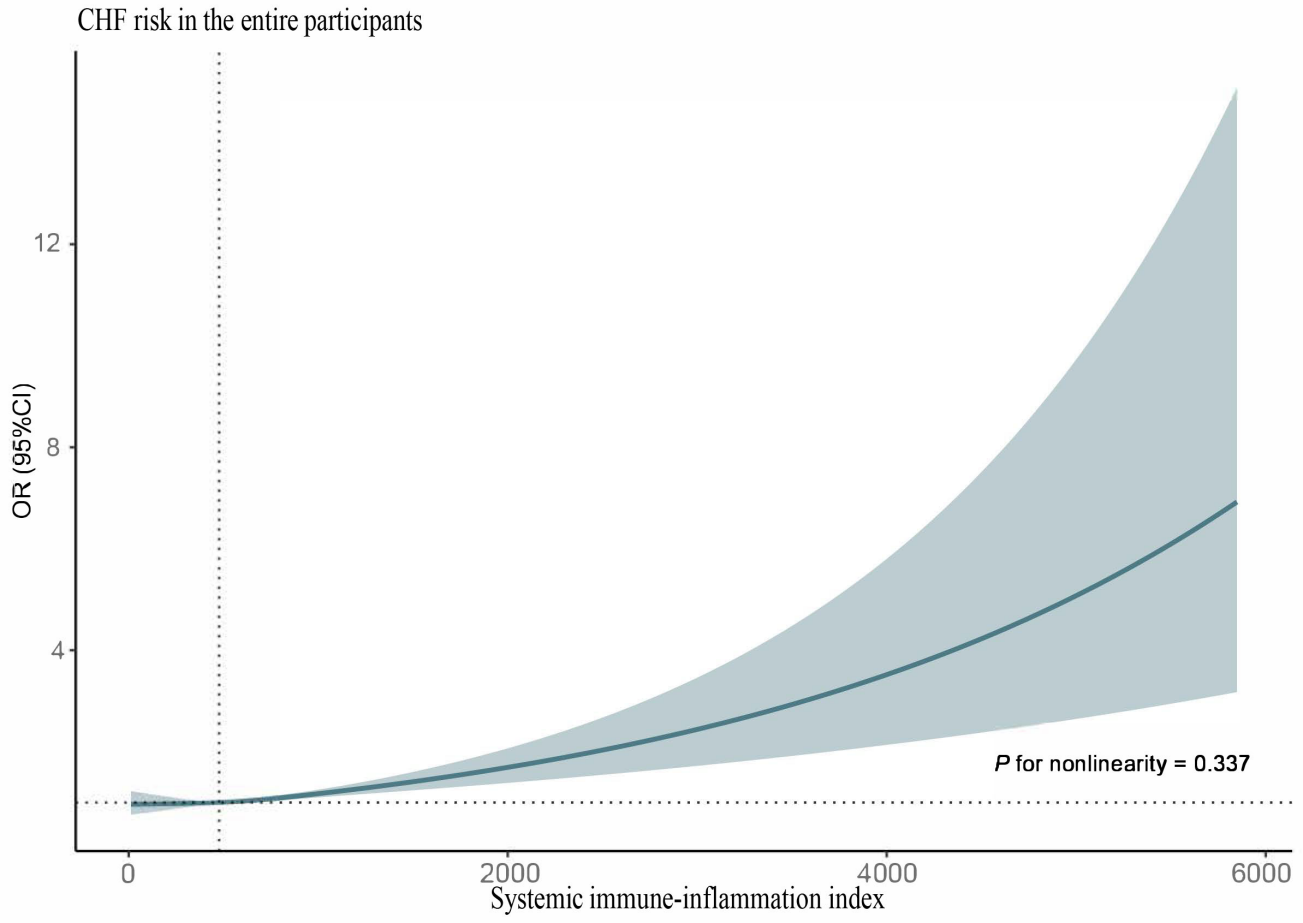
**RCS analysis for the correlation between SII levels and the risk of CHF**. Abbreviations: CHF, congestive heart failure; SII, systemic immune-inflammation index; RCS, restricted cubic spline; OR, odds ratio; CI, confidence interval.

Among participants enrolled in this study, there were 1927 (3.35%) participants diagnosed with CHF. Table 2 shows the logistic regression analysis for the association of SII with the prevalence of CHF. Participants with high SII levels had a significantly higher risk of CHF when compared to those with low SII levels (OR 1.24, 95% CI 1.05, 1.45). When analyzing SII as a continuous variable, RCS with the fully adjusted model showed that SII levels were positively associated with the risk of CHF (*p* for non-linearity = 0.645) (Fig. 2). Cox regression showed that with per one unit increasing in log-transformed SII, the risk of CHF was increased significantly by 72% (OR 1.72; 95% CI: 1.31, 2.25) (Table 2).

**Table 2.** **Logistic regression analysis for the risk of CHF according to SII among the whole people in NHANES**.

Model	Per one increase in log-transformed SII	OR (95% CI)			
	OR (95% CI)	Low	Median	High	*p* trend
Crude	2.55 (1.91, 3.39)	1.00	0.92 (0.76, 1.12)	1.48 (1.28, 1.70)	<0.001
Model 1	1.89 (1.46, 2.45)	1.00	0.91 (0.75, 1.11)	1.32 (1.14, 1.53)	<0.001
Model 2	1.66 (1.27, 2.15)	1.00	0.87 (0.71, 1.07)	1.21 (1.04, 1.41)	0.006
Model 3	1.72 (1.31, 2.25)	1.00	0.89 (0.73, 1.08)	1.24 (1.05, 1.45)	0.006

Model 1: adjusted for age, sex, and race/ethnicity; Model 2: further adjusted (from Model 1) for smoking status, physical activity, education level, family income to poverty ratio, and BMI; Model 3: further adjusted (from Model 2) for diabetes, hyperlipidemia, ASCVD, and hypertension. Abbreviations: CI, confidence interval; BMI, body mass index; CHF, congestive heart failure; SII, systemic immune-inflammation index; OR, odds ratio; NHANES, National Health and Nutrition Examination Survey; ASCVD, atherosclerotic cardiovascular disease.

### 3.3 Longitudinal Analysis: SII and the Prognosis in Patients with CHF

**Fig. 3.**
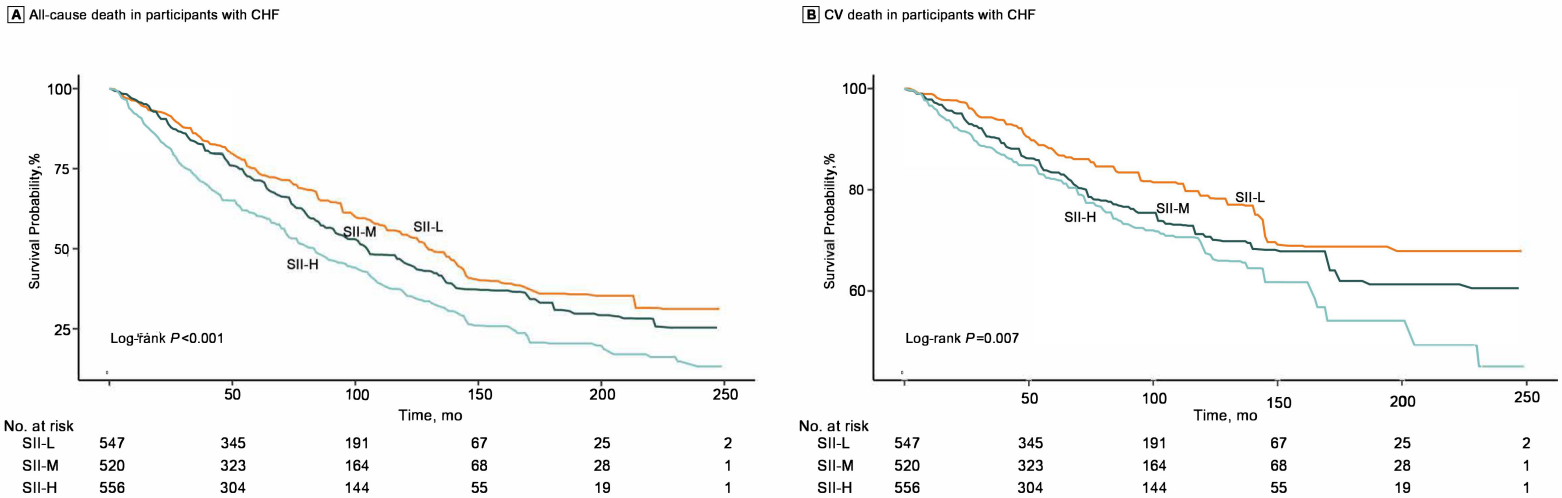
**K-M plots for the risk of all-cause death (A) and CV death (B) in groups of low SII, intermediate SII, and high SII among patients with CHF**. Abbreviations: SII-L, groups of low SII (<407.0); SII-M, groups of intermediate SII (407.0–672.8); SII-H, groups of high SII (>672.8); CHF, congestive heart failure; SII, systemic immune-inflammation index; K-M, Kaplan-Meier; CV, cardiovascular.

**Fig. 4.**
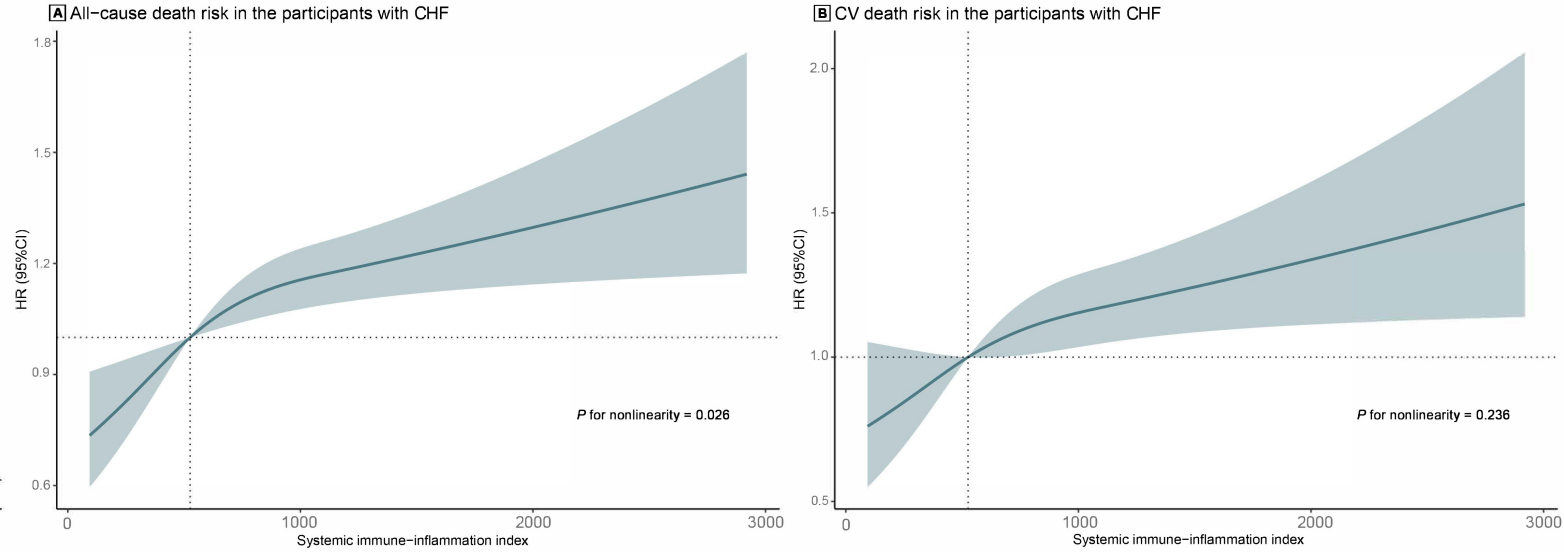
**RCS analysis for the correlation between SII levels and the risk of all-cause death (A) and CV death (B) in patients with CHF**. Abbreviations: CHF, congestive heart failure; SII, systemic immune-inflammation index; RCS, restricted cubic spline; CV, cardiovascular; HR, hazard ratio; CI, confidence interval.

During the median follow-up period of 5.4 years, there were 882 all-cause deaths including 379 CV deaths among CHF patients. Table 3 shows the Cox regression analysis for the association between SII and the risk of all-cause or CV death in CHF patients. Compared with the low SII group, patients with high SII levels had a significantly higher risk of all-cause (HR 1.44; 95% CI: 1.14, 1.81) and CV death (HR 1.31; 95% CI: 1.08, 1.58). K-M plots for three groups are presented in Fig. 3. Patients with high SII were at the highest risk of all-cause death (log-rank *p* < 0.001) and CV death (log-rank *p* = 0.007). In Fig. 4, RCS showed that SII levels were positively correlated with the risk of all-cause death (non-linear *p* = 0.026) and CV death (non-linear *p* = 0.236), and per one unit increase in log-transformed SII was associated with an increase of 69% in the risk of all-cause death (HR 1.69; 95% CI: 1.11, 2.57) and 43% for the risk of CV death (HR 1.43; 95% CI: 1.07, 1.92). The AUC for SII in predicting all-cause death in CHF patients was 0.61, and the AUC for predicting CV death was 0.57 (**Supplementary Fig. 2**).

**Table 3.** **Cox regression analysis for all-cause and cardiovascular mortality according to SII among patients with CHF in NHANES**.

Model		Per one increase in log-transformed SII	SII			
			HR (95% CI)			
		HR (95% CI)	Low	Median	High	*p* trend
All-cause death						
	Number of deaths/totals	882/1927	244/643	271/642	367/642	
	Crude	2.40 (1.60, 3.62)	1.00	1.20 (0.95, 1.53)	1.69 (1.32, 2.16)	<0.001
	Model 1	1.88 (1.27, 2.79)	1.00	1.10 (0.87, 1.37)	1.48 (1.18, 1.87)	<0.001
	Model 2	1.74 (1.14, 2.66)	1.00	1.11 (0.89, 1.38)	1.48 (1.17, 1.88)	<0.001
	Model 3	1.69 (1.11, 2.57)	1.00	1.07 (0.86, 1.33)	1.44 (1.14, 1.81)	0.002
Cardiovascular death						
	Number of deaths/totals	379/1927	98/643	125/642	156/642	
	Crude	1.48 (1.13, 1.93)	1.00	1.01 (0.82, 1.26)	1.34 (1.12, 1.61)	<0.001
	Model 1	1.49 (1.13, 1.96)	1.00	0.98 (0.79, 1.22)	1.32 (1.10, 1.58)	0.002
	Model 2	1.43 (1.07, 1.91)	1.00	0.99 (0.80, 1.24)	1.30 (1.08, 1.57)	0.003
	Model 3	1.43 (1.07, 1.92)	1.00	0.99 (0.79, 1.23)	1.31 (1.08, 1.58)	0.003

Model 1: adjusted for age, sex, and race/ethnicity; Model 2: further adjusted (from Model 1) for smoking status, physical activity, education level, family income to poverty ratio, and BMI; Model 3: further adjusted (from Model 2) for diabetes, hyperlipidemia, ASCVD, and hypertension. Abbreviations: HR, hazard ratio; CI, confidence interval; BMI, body mass index; CHF, congestive heart failure; SII, systemic immune-inflammation index; NHANES, National Health and Nutrition Examination Survey; ASCVD, atherosclerotic cardiovascular disease.

### 3.4 Subgroup and Sensitivity Analyses

Subgroup analyses for the association of SII levels with the prevalence of CHF in the whole population and the risk of all-cause/CV death are presented in **Supplementary Tables 3–5**. The results of the subgroup analyses were unchanged (all *p* for interaction > 0.05), except that high SII in the hypertensive population was associated with a greater risk of all-cause death.

Sensitivity analyses for the association between SII levels and the prevalence of CHF are listed in **Supplementary Table 6**. After excluding participants with incomplete data on BMI and PIR, the results did not change. **Supplementary Table 7** showed the sensitivity analyses for the association between SII levels and the risk of death in CHF patients. The results showed that high SII was still associated with an increased risk of all-cause and CV death after excluding participants who died within 90 days and provided incomplete data on BMI or PIR.

## 4. Discussion

This cross-sectional and longitudinal study analyzed 57,500 participants and found that high SII was significantly associated with a higher prevalence of CHF. In a further analysis of 1927 patients with CHF, the results showed that high SII was associated with an increased risk of all-cause death and CV death in patients with CHF. These findings reveal the potential value of SII in the prediction and prognosis of CHF and highlight the importance of SII in the prevention and management of CHF.

Although the relationship between SII and the incidence of CHF had not been previously investigated, the predictive value of SII has been demonstrated in other CVDs including CHD and various types of strokes. In 2021, a study was performed in a large population-based cohort (13,929 participants) with a median follow up of 8.28 years, which sought to evaluate the association of SII with the incidence of CVD (CHD and stroke) in Chinese adults. The results showed that higher levels of SII were associated with a higher risk of all types of strokes, suggesting SII was as a potential predictor for the incidence of stroke [9]. Similar conclusions were also reported in a cohort study with 45,809 subjects, which further investigated the impact of the dynamic status of SII and confirmed that the “increase pattern” of SII increased the risk of CVD by 38% [18]. A recent meta-analysis comprising 13 studies (152,996 participants) documented that high SII was associated with an increased risk of stroke, myocardial infarction, and peripheral arterial disease, the concept that SII was a valuable predictor for individuals with a high risk for CVD [19]. In the current study, we demonstrate the significant association of high SII with a high prevalence of CHF. This conclusion will need to be further validated in a large population-based cohort study or in randomized controlled trials.

In addition to its predictive value, the prognostic value of SII has also been widely studied in CVD. In a recent meta-analysis with 19 retrospective studies (18,609 stroke patients), it was determined that high SII was significantly associated with poor outcomes, high mortality, and a higher incidence of hemorrhagic transformation (HT) [10]. Zhao *et al*. [20] conducted a cohort study of 3561 patients with three-vessel CHD to investigate the relationship between SII levels and prognosis in CHD. This revealed that high SII was independently associated with a high risk of major adverse cerebrovascular and cardiovascular events. The addition of SII levels to the “traditional risk factor” prediction model was shown to significantly improve its sensitivity and specificity in predicting long-term prognosis in patients with CHD. A recent cohort study with 717 CHF patients with renal dysfunction suggested that high SII levels significantly increased the risk of all-cause death and major adverse cardiovascular events [21]. Similar conclusions were also demonstrated in another cohort study which focused on critically ill patients with CHF based on the Medical Information Mart for Intensive Care III (MIMIC III) database [22]. Although SII has been demonstrated as a prognostic marker in patients with CHF, the population analyzed in previous studies was only limited to those with renal dysfunction or critical illness. In the current study, we focused for the first time on general CHF patients and validated the prognostic value of SII in these patients, further supporting that SII was a prognostic biomarker in CHF patients.

The pivotal role of inflammation in the pathogenesis and development of CHF has been widely discussed [23]. Previous study has determined that high inflammatory burden contributed to cardiomyocyte necrosis and interstitial fibrosis, which subsequently led to altered cardiac contractility and cardiac dysfunction [24]. SII is a novel biomarker evaluating the systemic inflammatory burden and the components of SII: platelets, leukocytes, and neutrophils, all of which have been shown to contribute to cardiotoxicity. High levels of platelets increase thrombosis which leads to endothelial injury and atherosclerosis [25]. The activated platelet has been shown to further recruit leukocytes and neutrophils via P-selectin and β_2_/β_3_-integrin receptors, resulting in an increase in local inflammation which results in cardiomyocyte necrosis [26, 27]. These mechanisms are potential explanations for the significant association between SII and CHF, however, the precise mechanisms responsible for the increased SII in CHF patients has not fully been elucidated. Further research is needed to study this topic.

In addition to SII, several biomarkers such as pentraxin-3 and receptors for advanced glycation endproducts have also been found to be specific markers to evaluate the inflammatory burden in patients with HF [28]. However, they are not commonly available in clinical practice and are influenced by several physiological conditions. Compared to these markers, the components of SII are much more readily available and cost-effective as they can be obtained from a routine blood test, and are more easily monitored. Since our study found that high SII was related to the high incidence of CHF, regular cardiology tests such as an echocardiogram is recommended for individuals with high SII. CHF patients with high SII will require closer follow-up and monitoring from cardiologists.

The main strengths of this study included demonstrating the predictive value of SII on the incidence of CHF and its prognostic value in patients with CHF. However, this study has a few limitations. First, the SII data was only collected at baseline and the lack of data on dynamic changes in SII might bias the estimation of the relationship between SII and prognosis. Second, the diagnosis of CHF in this study depended on the self-reported questionnaires without laboratory results and cardio-imaging and without further evaluation by cardiologists. This may have resulted in missing some patients with CHF, potentially underestimating the prevalence of CHF. The type and extent of CHF could not be elaborated, limiting further sensitivity analyses. Third, the association between SII levels and the incidence of CHF is based on cross-sectional data with a low level of evidence. Therefore, this association needs to be validated in future cohort studies or randomized controlled studies. Fourth, we did not exclude participants who had infection, hematologic disease, and peroral steroid treatment because NHANES did not provide related information for each subject. These conditions might significantly contribute to the effect of the level of SII. Fifth, the sample size of this study is not sufficiently large, and the population is not sufficiently diverse. We only included the American population, with the majority being non-Hispanic whites and non-Hispanic blacks, which affects the generalizability of the results. Sixth, the duration of follow-up varied widely, ranging from 20 years to a few months. Some participants, who were recently enrolled in the program may have been inadequately followed up, potentially reducing event rates and underestimating the association between SII and CHF. Although we excluded participants who died within 90 days, the results were still significant. Finally, a common issue with retrospective studies is the presence of uncorrected covariates that may impact outcomes. Further randomized, controlled studies are needed to confirm these findings.

## 5. Conclusions

In conclusion, this cross-sectional and longitudinal study documented that SII was a potential biomarker with great value in predicting the incidence of CHF and the prognosis in patients with CHF. These findings underscore the potential role of SII in the prevention and management of CHF, suggesting that monitoring SII could be crucial for early detection and intervention. Further investigation is needed to confirm our study and elucidate the specific roles of SII in the pathogenesis and development of CHF.

## Availability of Data and Materials

All data are available at NHANES website https://www.cdc.gov/nchs/nhanes/index.htm.

## Supplementary Material

Supplementary material associated with this article can be found, in the online version, at https://doi.org/10.31083/j.rcm2511417.
