# Supplementary material for: The Predictive and Prognostic Value of the Systemic Immune-Inflammation Index for Congestive Heart Failure
Source: Rev Cardiovasc Med. 2024 Nov 21;25(11):417. doi: 10.31083/j.rcm2511417 (PMC11607486; doi:10.31083/j.rcm2511417)
Supplement: Supplementary file 1 [file 2153-8174-25-11-417-s1.docx]

Supplementary Table 1. Baseline characteristics of the entire participants grouped by CHF in NHANES.

| Characteristics | Total (N=57500) | CHF | |  |
| --- | --- | --- | --- | --- |
|  |  | No | Yes | *P* value |
| Age (years) | 47.46±0.19 | 46.99±0.18 | 66.31±0.40 | < 0.001 |
| Sex, n (%) |  |  |  | < 0.001 |
| Male | 27667(48.12) | 26582(47.89) | 1085(53.20) |  |
| Female | 29833(51.88) | 28991(52.11) | 842(46.80) |  |
| Race/ethnicity, n (%) |  |  |  | < 0.001 |
| Non-Hispanic White | 24853(43.22) | 23824(67.30) | 1029(72.75) |  |
| Non-Hispanic Black | 12151(21.13) | 11677(10.78) | 474(14.08) |  |
| Mexican American | 9676(16.83) | 9489(8.53) | 187(3.81) |  |
| Others | 10820(18.82) | 10583(13.39) | 237(9.36) |  |
| Education level, n (%) |  |  |  | < 0.001 |
| Less than high school | 14892(25.9) | 14172(15.56) | 720(28.19) |  |
| High school or equivalent | 13367(23.25) | 12880(23.98) | 487(28.38) |  |
| College or above | 29241(50.85) | 28521(60.45) | 720(43.43) |  |
| Family income to poverty ratio, n (%) | |  |  | 0.003 |
| <1 | 10694(20.52) | 10269(13.86) | 425(18.90) |  |
| ≥1 & <3 | 21976(42.18) | 21041(35.47) | 935(51.73) |  |
| ≥3 | 19434(37.3) | 19043(50.67) | 391(29.37) |  |
| Unknown | 10694(20.52) | 10269(13.86) | 425(18.90) |  |
| Smoking status, n (%) |  |  |  | < 0.001 |
| Never | 31566(54.9) | 10921(57.26) | 10621(55.58) |  |
| Former | 14185(24.67) | 4611(24.85) | 4661(24.30) |  |
| Current | 11749(20.43) | 3636(17.89) | 3883(20.12) |  |
| BMI (kg/m^2^), n (%) |  |  |  | < 0.001 |
| <25.0 | 16378(28.98) | 16038(30.11) | 340(17.44) |  |
| 25.0–29.9 | 18995(33.61) | 18466(33.23) | 529(26.70) |  |
| ≥30.0 | 21151(37.42) | 20193(36.66) | 958(55.86) |  |
| Physical activity, n (%) |  |  |  | < 0.001 |
| Sedentary | 15904(27.66) | 14949(21.34) | 955(47.07) |  |
| Insufficient | 11877(20.66) | 11532(18.74) | 345(16.05) |  |
| Moderate | 6566(11.42) | 6391(11.65) | 175(11.05) |  |
| High | 23153(40.27) | 22701(48.27) | 452(25.83) |  |
| ASCVD, n (%) | 5910(10.28) | 4599(6.76) | 1311(67.07) | < 0.001 |
| Diabetes, n (%) | 10287(17.89) | 9352(13.09) | 935(47.05) | < 0.001 |
| Hyperlipidemia, n (%) | 40912(71.15) | 39257(69.40) | 1655(87.23) | < 0.001 |
| Hypertension, n (%) | 23837(41.46) | 22252(35.81) | 1585(80.27) | < 0.001 |
| SII | 555.54±2.92 | 552.90± 2.86 | 660.42±15.53 | < 0.001 |

Data are presented as weighted means ± SEs for continuous variables and unweighted numbers (weighted percentages) for categorical variables.

Abbreviations: ASCVD, atherosclerotic cardiovascular disease; BMI, body mass index; CHF, congestive heart failure; SII, systemic immune-inflammation index.

Supplementary Table 2. Baseline characteristics of the CHF participants grouped by SII in NHANES.

| Characteristics | Total (N=1927) | **SII** | | |  |
| --- | --- | --- | --- | --- | --- |
|  |  | Low (N=575) | Median (N=553) | High (N=799) | *P* value |
| Age (years) | 66.31±0.40 | 64.45±0.86 | 66.33±0.72 | 67.51±0.55 | 0.02 |
| Sex, n (%) |  |  |  |  | 0.25 |
| Male | 1085(56.31) | 345(56.78) | 303(53.68) | 437(50.57) |  |
| Female | 842(43.69) | 230(43.22) | 250(46.32) | 362(49.43) |  |
| Race/ethnicity, n (%) |  |  |  |  | < 0.001 |
| Non-Hispanic White | 1029(53.4) | 239(63.24) | 293(73.10) | 497(78.70) |  |
| Non-Hispanic Black | 474(24.6) | 196(21.32) | 137(13.58) | 141(9.70) |  |
| Mexican American | 187(9.7) | 50(3.54) | 61(4.33) | 76(3.64) |  |
| Others | 237(12.3) | 90(11.90) | 62(8.99) | 85(7.95) |  |
| Education level, n (%) |  |  |  |  | 0.29 |
| Less than high school | 720(37.36) | 222(27.77) | 195(26.49) | 303(29.58) |  |
| High school or equivalent | 487(25.27) | 141(27.21) | 125(25.76) | 221(30.85) |  |
| College or above | 720(37.36) | 212(45.02) | 233(47.75) | 275(39.57) |  |
| Family income to poverty ratio, n (%) | |  |  |  | 0.23 |
| <1 | 425(22.06) | 133(17.82) | 120(15.81) | 172(18.33) |  |
| ≥1 & <3 | 935(48.52) | 274(47.75) | 269(47.76) | 392(47.86) |  |
| ≥3 | 391(20.29) | 120(26.29) | 122(31.30) | 149(24.97) |  |
| Unknown | 176(9.13) | 48(8.14) | 42(5.13) | 86(8.85) |  |
| Smoking status, n (%) |  |  |  |  | 0.84 |
| Never | 755(39.18) | 237(38.03) | 223(39.19) | 295(37.12) |  |
| Former | 821(42.61) | 230(44.58) | 240(43.94) | 351(42.98) |  |
| Current | 351(18.21) | 108(17.39) | 90(16.87) | 153(19.90) |  |
| BMI (kg/m^2^), n (%) |  |  |  |  | 0.93 |
| <25.0 | 340(17.64) | 102(16.20) | 92(17.21) | 146(16.65) |  |
| 25.0–29.9 | 629(32.64) | 179(30.41) | 186(27.90) | 264(30.83) |  |
| ≥30.0 | 958(49.71) | 294(53.39) | 275(54.90) | 389(52.52) |  |
| Physical activity, n (%) |  |  |  |  | 0.72 |
| Sedentary | 955(49.56) | 266(43.94) | 273(48.21) | 416(48.35) |  |
| Insufficient | 345(17.9) | 110(16.12) | 107(15.56) | 128(16.32) |  |
| Moderate | 175(9.08) | 54(10.70) | 49(12.66) | 72(10.23) |  |
| High | 452(23.46) | 145(29.24) | 124(23.56) | 183(25.10) |  |
| ASCVCD, n (%) | 1311(68.03) | 449(69.88) | 431(66.74) | 431(64.72) | 0.38 |
| Diabetes, n (%) | 935(48.52) | 268(42.86) | 268(48.89) | 399(48.56) | 0.22 |
| Hyperlipidemia, n (%) | 1655(85.88) | 489(85.04) | 482(88.37) | 684(87.91) | 0.41 |
| Hypertension, n (%) | 1585(82.25) | 474(77.98) | 448(79.56) | 663(82.23) | 0.39 |
| eGFR (mL/min/1.73 m^2^) | 68.74±0.78 | 73.14±1.37 | 68.93±1.60 | 65.77±1.08 | < 0.001 |
| Waist circumference (cm) | 109.41±0.66 | 106.91±0.93 | 110.89±1.15 | 110.07±0.89 | 0.01 |

Data are presented as weighted means ± SEs for continuous variables and unweighted numbers (weighted percentages) for categorical variables.

Abbreviations: ASCVD, atherosclerotic cardiovascular disease; BMI, body mass index; CHF, congestive heart failure; SII, systemic immune-inflammation index.

Supplementary Table 3. Subgroup analyses of the associations between SII and CHF among the entire participants.

| Subgroup | No. HF/Total | OR (95% CI) | | | *P* for interaction |
| --- | --- | --- | --- | --- | --- |
|  |  | Low | Median | High |  |
| Age (years) |  |  |  |  | 0.075 |
| <60 | 434/37759 | 1.00 | 0.78(0.52, 1.16) | 0.91(0.65, 1.29) |  |
| ≥60 | 1493/19741 | 1.00 | 0.95(0.78, 1.16) | 1.43(1.20, 1.69) |  |
| Sex |  |  |  |  | 0.952 |
| Male | 1085/27667 | 1.00 | 0.88(0.69, 1.13) | 1.24(0.98, 1.57) |  |
| Female | 842/29833 | 1.00 | 0.91(0.69, 1.19) | 1.26(1.00, 1.58) |  |
| Race/ethnicity |  |  |  |  | 0.509 |
| Non-Hispanic people | 1503/37004 | 1.00 | 1.00(0.79, 1.26) | 1.36(1.12, 1.66) |  |
| Other | 424/20496 | 1.00 | 0.86(0.56, 1.31) | 0.95(0.65, 1.38) |  |
| Smoking status |  |  |  |  | 0.772 |
| Never | 755/31566 | 1.00 | 0.94(0.67, 1.31) | 1.33(1.03, 1.73) |  |
| Former/Current | 1172/25934 | 1.00 | 0.86(0.69, 1.07) | 1.19(0.99, 1.44) |  |
| BMI, kg/m^2^ |  |  |  |  | 0.108 |
| <30 | 969/36349 | 1.00 | 0.91(0.70, 1.17) | 1.2(0.99, 1.46) |  |
| ≥30 | 958/21151 | 1.00 | 0.87(0.65, 1.15) | 1.25(0.97, 1.60) |  |
| Physical activity |  |  |  |  | 0.910 |
| Sedentary/Insufficient | 1300/27781 | 1.00 | 0.87(0.67, 1.14) | 1.19(0.98, 1.43) |  |
| Moderate/High | 627/29719 | 1.00 | 0.89(0.66, 1.21) | 1.33(1.03, 1.72) |  |
| ASCVD |  |  |  |  | 0.210 |
| Yes | 1311/5911 | 1.00 | 0.92(0.74, 1.13) | 1.18(0.98, 1.42) |  |
| No | 616/51589 | 1.00 | 0.85(0.61, 1.18) | 1.31(0.97, 1.76) |  |
| Hyperlipidemia |  |  |  |  |  |
| Yes | 1655/40912 | 1.00 | 0.98(0.77, 1.23) | 1.31(1.08, 1.60) | 0.606 |
| No | 272/16588 | 1.00 | 0.77(0.43, 1.36) | 1.04(0.66, 1.64) |  |
| Hypertension |  |  |  |  |  |
| Yes | 1585/23837 | 1.00 | 0.96(0.76, 1.21) | 1.34(1.10, 1.63) | 0.666 |
| No | 342/33663 | 1.00 | 0.91(0.59, 1.40) | 1.05(0.70, 1.57) |  |
| Diabetes |  |  |  |  | 0.382 |
| Yes | 935/10287 | 1.00 | 1.00(0.77, 1.30) | 1.43(1.14, 1.79) |  |
| No | 992/472123 | 1.00 | 0.82(0.62, 1.06) | 1.12(0.90, 1.38) |  |

All the Models were adjusted for age, sex, race/ethnicity, smoking status, physical activity, education level, family income to poverty ratio, BMI, diabetes, dyslipidemia, ASCVD, and hypertension.

Abbreviations: HRs, hazard ratios; CIs, confidence intervals; BMI, body mass index, ASCVD, atherosclerotic cardiovascular disease.

Supplementary Table 4. Subgroup analyses of the associations between SII and all-cause mortality among patients with CHF.

| Subgroup | No. All-cause death/Total | HR (95% CI) | | | *P* for interaction |
| --- | --- | --- | --- | --- | --- |
|  |  | Low | Median | High |  |
| Age (years) |  |  |  |  | 0.334 |
| <60 | 109/434 | 1.00 | 1.03(0.58, 1.83) | 2.12(1.09, 4.12) |  |
| ≥60 | 773/1493 | 1.00 | 1.07(0.84, 1.36) | 1.27(1.00, 1.62) |  |
| Sex |  |  |  |  | 0.582 |
| Male | 506/1085 | 1.00 | 1.02(0.75, 1.38) | 1.33(0.98, 1.79) |  |
| Female | 376/842 | 1.00 | 1.16(0.85, 1.59) | 1.55(1.12, 2.14) |  |
| Race/ethnicity |  |  |  |  | 0.523 |
| Non-Hispanic people | 730/1503 | 1.00 | 1.08(0.85, 1.38) | 1.42(1.11, 1.80) |  |
| Other | 152/424 | 1.00 | 0.91(0.48, 1.72) | 2.15(1.15, 4.02) |  |
| Smoking status |  |  |  |  | 0.333 |
| Never | 339/755 | 1.00 | 0.91(0.66, 1.27) | 1.25(0.85, 1.84) |  |
| Former/Current | 543/1172 | 1.00 | 1.19(0.86, 1.64) | 1.66(1.27, 2.18) |  |
| BMI, kg/m^2^ |  |  |  |  | 0.117 |
| <30 | 522/969 | 1.00 | 1.18(0.89, 1.57) | 1.36(1.01, 1.83) |  |
| ≥30 | 360/958 | 1.00 | 0.98(0.67, 1.43) | 1.63(1.14, 2.33) |  |
| Physical activity |  |  |  |  | 0.805 |
| Sedentary/Insufficient | 704/1300 | 1.00 | 1.09(0.86, 1.38) | 1.46(1.10, 1.93) |  |
| Moderate/High | 178/627 | 1.00 | 0.95(0.60, 1.53) | 1.48(0.94, 2.32) |  |
| ASCVD |  |  |  |  | 0.292 |
| Yes | 626/1311 | 1.00 | 1.15(0.87, 1.51) | 1.41(1.06, 1.88) |  |
| No | 256/616 | 1.00 | 0.98(0.66, 1.46) | 1.52(1.00, 2.32) |  |
| Hyperlipidemia |  |  |  |  | 0.248 |
| Yes | 758/1655 | 1.00 | 1.08(0.84, 1.38) | 1.53(1.19, 1.95) |  |
| No | 124/272 | 1.00 | 0.96(0.52, 1.78) | 1.14(0.62, 2.09) |  |
| Hypertension |  |  |  |  | <0.001 |
| Yes | 713/1585 | 1.00 | 1.14(0.90, 1.46) | 1.74(1.34, 2.25) |  |
| No | 169/342 | 1.00 | 0.87(0.52, 1.47) | 0.82(0.53, 1.27) |  |
| Diabetes |  |  |  |  | 0.055 |
| Yes | 437/935 | 1.00 | 1.26(0.90, 1.76) | 1.87(1.35, 2.59) |  |
| No | 445/992 | 1.00 | 1.02(0.77, 1.35) | 1.18(0.89, 1.57) |  |

All the Models were adjusted for age, sex, race/ethnicity, smoking status, physical activity, education level, family income to poverty ratio, BMI, diabetes, dyslipidemia, ASCVD, and hypertension.

Abbreviations: HRs, hazard ratios; CIs, confidence intervals; BMI, body mass index, ASCVD, atherosclerotic cardiovascular disease.

Supplementary Table 5. Subgroup analyses of the associations between SII and CV mortality among patients with CHF.

| Subgroup | No. All-cause death/Total | HR (95% CI) | | | *P* for interaction |
| --- | --- | --- | --- | --- | --- |
|  |  | Low | Median | High |  |
| Age (years) |  |  |  |  | 0.084 |
| <60 | 44/434 | 1.00 | 1.85(0.84, 4.10) | 4.00(1.35, 11.84) |  |
| ≥60 | 335/1493 | 1.00 | 1.12(0.81, 1.56) | 1.21(0.82, 1.76) |  |
| Sex |  |  |  |  | 0.534 |
| Male | 215/1085 | 1.00 | 1.47(0.97, 2.24) | 1.53(1.00, 2.34) |  |
| Female | 164/842 | 1.00 | 1.01(0.63, 1.65) | 1.30(0.79, 2.13) |  |
| Race/ethnicity |  |  |  |  | 0.389 |
| Non-Hispanic people | 318/1503 | 1.00 | 1.23(0.88, 1.71) | 1.37(0.96, 1.95) |  |
| Other | 61/424 | 1.00 | 1.20(0.46, 3.16) | 2.43(0.99, 5.96) |  |
| Smoking status |  |  |  |  | 0.958 |
| Never | 169/755 | 1.00 | 1.14(0.71, 1.85) | 1.37(0.77, 2.43) |  |
| Former/Current | 210/1172 | 1.00 | 1.32(0.81, 2.15) | 1.54(1.01, 2.35) |  |
| BMI, kg/m^2^ |  |  |  |  | 0.680 |
| <30 | 228/969 | 1.00 | 1.11(0.68, 1.82) | 1.30(0.84, 2.02) |  |
| ≥30 | 151/958 | 1.00 | 1.23(0.71, 2.14) | 1.67(1.00, 2.80) |  |
| Physical activity |  |  |  |  | 0.975 |
| Sedentary/Insufficient | 308/1300 | 1.00 | 1.17(0.83, 1.65) | 1.41(0.92, 2.17) |  |
| Moderate/High | 71/627 | 1.00 | 1.48(0.74, 2.96) | 1.45(0.69, 3.06) |  |
| ASCVD |  |  |  |  | 0.700 |
| Yes | 277/1311 | 1.00 | 1.36(0.93, 1.98) | 1.53(0.96, 2.44) |  |
| No | 102/616 | 1.00 | 1.00(0.50, 2.01) | 1.20(0.58, 2.48) |  |
| Hyperlipidemia |  |  |  |  | 0.803 |
| Yes | 323/1655 | 1.00 | 1.19(0.83, 1.70) | 1.43(0.97, 2.10) |  |
| No | 56/272 | 1.00 | 1.38(0.46, 4.18) | 2.14(0.81, 5.62) |  |
| Hypertension |  |  |  |  | 0.233 |
| Yes | 312/1585 | 1.00 | 1.17(0.82, 1.67) | 1.52(1.02, 2.28) |  |
| No | 67/342 | 1.00 | 1.72(0.76, 3.88) | 1.34(0.64, 2.80) |  |
| Diabetes |  |  |  |  | 0.503 |
| Yes | 189/935 | 1.00 | 1.16(0.72, 1.85) | 1.25(0.77, 2.02) |  |
| No | 190/992 | 1.00 | 1.43(0.91, 2.24) | 1.69(1.00, 2.83) |  |

All the Models were adjusted for age, sex, race/ethnicity, smoking status, physical activity, education level, family income to poverty ratio, BMI, diabetes, dyslipidemia, ASCVD, and hypertension.

Abbreviations: HRs, hazard ratios; CIs, confidence intervals; BMI, body mass index, ASCVD, atherosclerotic cardiovascular disease.

Supplementary Table 6. Sensitivity Analyses of the associations between SII and the risk of CHF among the whole people in NHANES.

| Analysis | OR (95% CI) | | | *P* trend |
| --- | --- | --- | --- | --- |
|  | Low | Median | High |  |
| Excluding non-Hispanic Black participants (N=45349) | 1.00 | 0.95(0.76, 1.19) | 1.35(1.11, 1.63) | 0.001 |
| Excluding participants with missing data on BMI (N=56524) | 1.00 | 0.91(0.75, 1.12) | 1.28(1.08, 1.51) | 0.003 |
| Excluding participants with missing data on PIR (N=52104) | 1.00 | 0.95(0.76, 1.18) | 1.28(1.07, 1.53) | 0.005 |

All the Models were adjusted for age, sex, race/ethnicity, smoking status, physical activity, education level, family income to poverty ratio, BMI, diabetes, dyslipidemia, ASCVD, and hypertension.

Abbreviations: HRs, hazard ratios; CIs, confidence intervals; BMI, body mass index, ASCVD, atherosclerotic cardiovascular disease, PIR, family income to poverty ratio.

Supplementary Table 7. Sensitivity Analyses of the associations between SII and all-cause/CV mortality among CHF patients.

| Analysis | HR (95% CI) | | | *P* trend |
| --- | --- | --- | --- | --- |
|  | Low | Median | High |  |
| Excluding participants who died within 90 days of follow-up (N=1817) |  |  |  |  |
| All-cause mortality | 1.00 | 1.07(0.86, 1.33) | 1.46(1.16, 1.85) | 0.001 |
| CV mortality | 1.00 | 1.21(0.90, 1.64) | 1.43(1.01, 2.02) | 0.050 |
| Excluding participants with missing data on BMI (N=1827) |  |  |  |  |
| All-cause mortality | 1.00 | 0.98(0.75, 1.27) | 1.32(1.04, 1.68) | 0.013 |
| CV mortality | 1.00 | 1.06(0.75, 1.50) | 1.22(0.87, 1.70) | 0.003 |
| Excluding participants with missing data on PIR (N=1751) |  |  |  |  |
| All-cause mortality | 1.00 | 0.97(0.75, 1.26) | 1.32(1.02, 1.69) | 0.022 |
| CV mortality | 1.00 | 1.02(0.72, 1.45) | 1.20(1.03, 1.72) | 0.023 |

All the Models were adjusted for age, sex, race/ethnicity, smoking status, physical activity, education level, family income to poverty ratio, BMI, diabetes, dyslipidemia, ASCVD, and hypertension.

Abbreviations: HRs, hazard ratios; CIs, confidence intervals; BMI, body mass index, ASCVD, atherosclerotic cardiovascular disease, PIR, family income to poverty ratio.


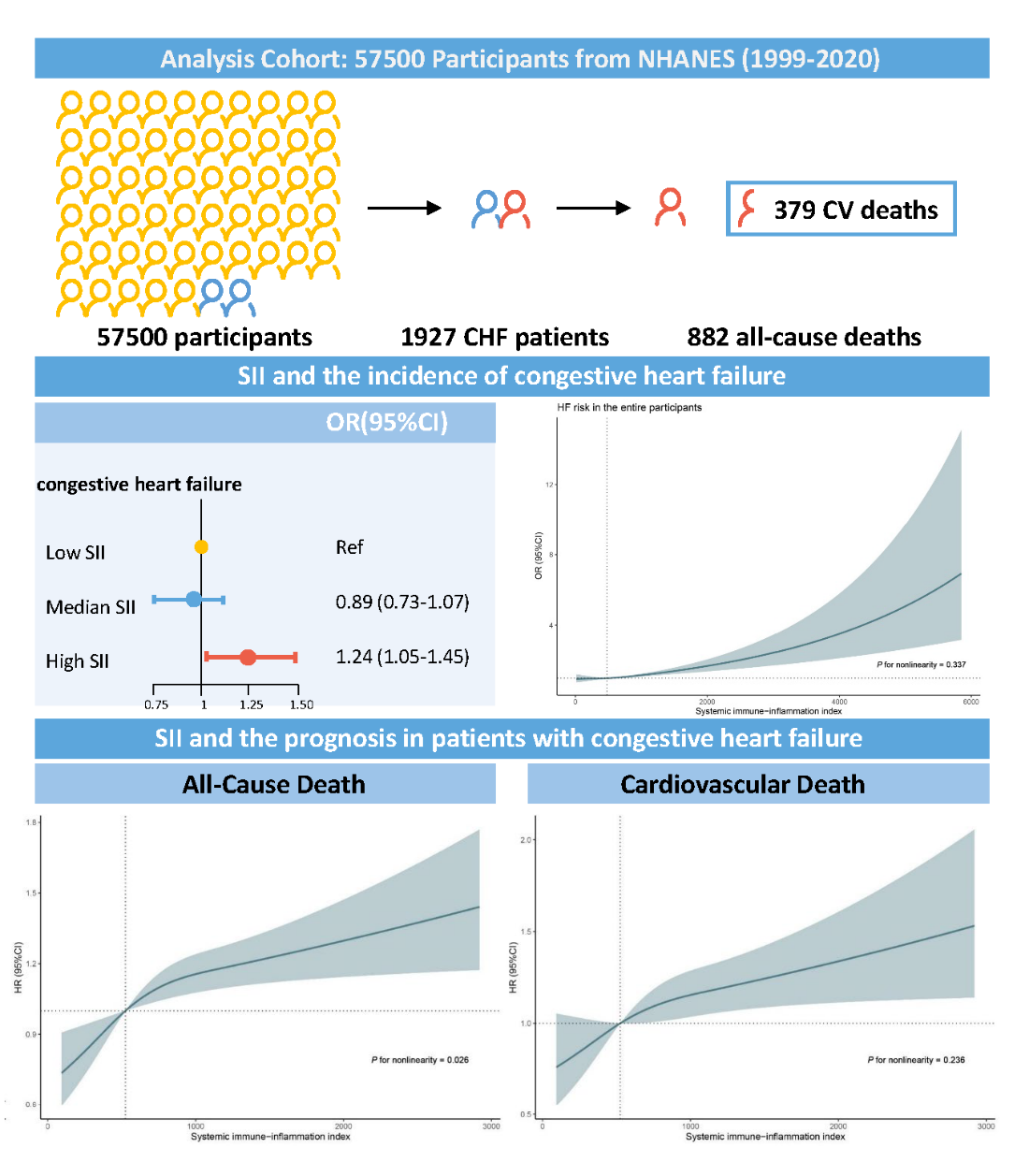


**Supplementary Fig. 1.** **Graphic Abstract.** Abbreviations: NAHNES, National Health and Nutrition Examination Survey; CV, cardiovascular; SII, systemic immune-inflammation index.

**
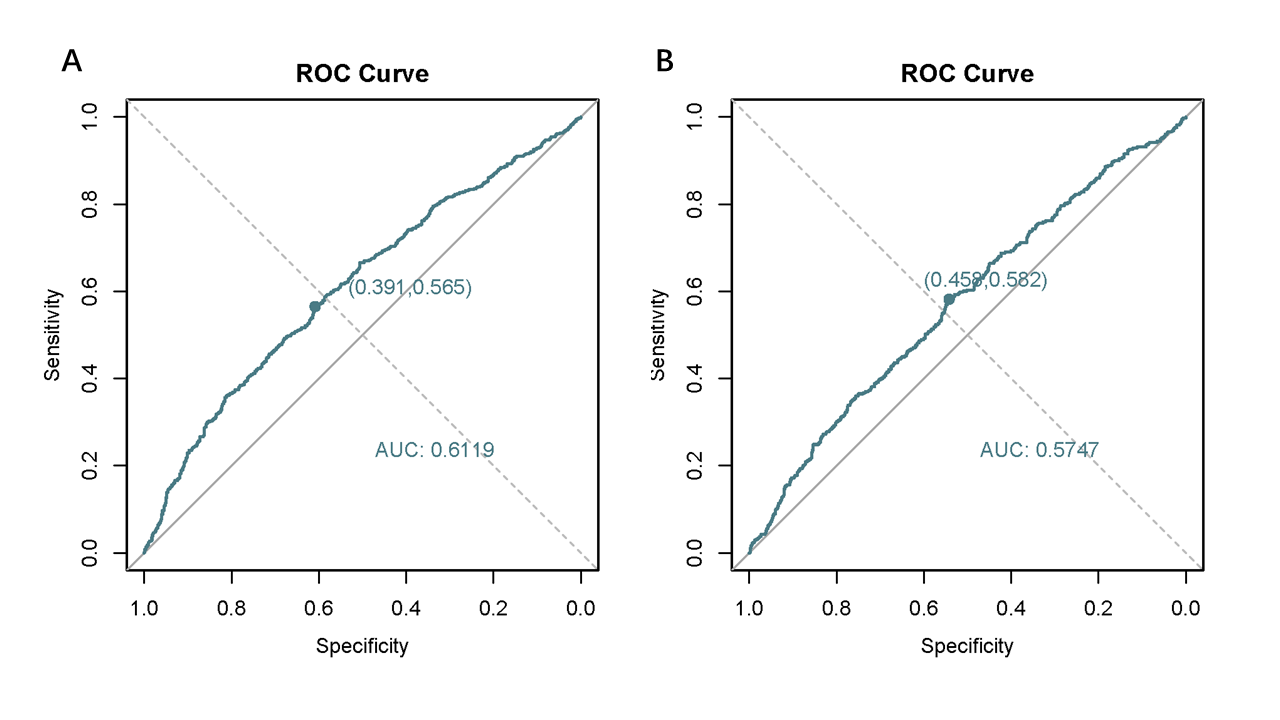
**

**Supplementary Fig. 2. ROC curves for SII levels predicting prognosis in CHF patients.** Abbreviations: CHF, congestive heart failure; SII, systemic immune-inflammation index; ROC, receiver operating characteristic. (A) All-cause death; (B) Cardiovascular death.
